# Therapeutic Notes

**Published:** 1895-06-01

**Authors:** 


### THERAPEUTIC NOTES.

### CHLORALOSE.

This hypnotic has now been in use for a considerable period, and it will be useful to recapitulate its properties. Chloralose is a crystalline body having the formula, $C_8 H_{11} Cl_9 O_6$, and is prepared by acting on anhydrous chloral ($C_2 H Cl_3 O$) with glucose. It is slightly soluble in water and in ether, more so in hot water and in alcohol. The aqueous solution does not reduce Fehling's solution even when boiling; dilute acids do not decompose it, but on warming with alkalis a brown colour develops. By oxidisation chloralose is converted into chloralic acid $C_7 H_9 Cl_3 O_6$. The dose is three to twenty grains, and it may be given in powder or in solution. Its taste is bitter. Experiments made with chloralose on frogs show that minute doses increase the reflex excitability of the spinal cord, while larger doses depress the whole central nervous system, the animal finally lying as if quite dead, although on examination its heart is found to be beating regularly and strongly, and may continue to do so for two hours or more after respiration and voluntary movements have ceased. In the lower mammalia small doses increase the excitability of the cord, larger amounts lessen the power of voluntary motion and sensibility to pain, and induce sleep. If the sleep be not profound, tonic and clonic convulsions are a prominent feature. Dogs show all the effects typically. In these animals sleep is preceded by a period of excitement, during which the animal staggers about as if intoxicated and is insensible to its surroundings, its reflexes are exaggerated and the sense of pain is abolished. Convulsions occur if the animal does not fall deeply asleep. Temperature is markedly lowered, but the rate of the heart and blood-pressure are affected only by large doses. Small or medium doses do not apparently depress the circulatory system.

In man the action of chloralose is essentially hypnotic. With doses of four grains only a distinctly soothing influence is observable; with eight grains the hypnotic effect is uncertain, but with fifteen to twenty grains a profound sleep is brought on which lasts many hours. Nevertheless in very slight degrees of insomnia the soothing effects of three to six grains are often sufficient to bring on sleep. The time elapsing between the administration of the drug and the onset of sleep varied from a half to three and a half hours, but half an hour or a little more is the usual interval. In most cases sleep comes on gradually, preceded by a pleasant sense of drowsiness, but in others it comes on suddenly while the patient is engaged in eating or talking. The sleep is usually profound, the patient being insensible to all slight external stimuli, such as touching the cornea, loud noises, &c. Frequently muscular twitchings, tremors, dizziness, disturbance of speech, and other signs of motor and sensory affection of the nervous system are observed. When large doses are given, convulsive attacks without sleep have been observed. After waking the patient may suffer from headache or hebetude for a time.

As a therapeutic agent its soothing and hypnotic effects are of great use in properly-chosen cases. In nervous insomnia, in cerebral excitement, in the insomnia and pain of late cardiac disease it has been found extremely valuable. But it is of little use in sleeplessness due to pain or to delirium tremens, and it aggravates the inco-ordination and tremors of organic nervous diseases.

Flemming, using mostly doses of two to six grains, gives a more favourable account of its hypnotic action. He found that sleep came on in from twenty to sixty minutes, and lasted from four to ten hours or longer, and no unpleasant effects of any kind were observed either before, during, or after the sleep. After considerable experience, he thinks that benefit may be expected from the use of chloralose in all forms of functional insomnia, and in sleeplessness from psychical excitement, hysteria, neurasthenia, overwork, or cardiac irritability, and in epilepsy and somnambulism. In other conditions it is of little value. Probably the use of small doses prevented Flemming from seeing ill-effects which have been recorded by other observers. L'Hoest, using similar or slightly larger doses than Flemming, had an exactly similar experience as regards efficacy and absence of poisonous effects. His experience was confined to asylum patients, and he pronounces the result to be "absolutely favourable." Sacaze, after discovering accidentally that it influences favourably the night sweats of phthisis, employs it now as follows: about the time the patient wishes to go to sleep one grain is given; if sleep fails to come on the dose is repeated every half-hour until four cachets have been given. When the temperature rises at night a small dose of sulphate of quinine may be added to the cachet with marked benefit to the patient. Sacaze is of opinion that it is fully as efficacious as atropine and other remedies used in night sweating. Owing to its stimulant effect on the spinal cord it should be avoided in cases where there is a tendency to hyper-excitability, as it is then apt to produce spasms, which, however, are never very severe. Cases of collapse with great nervous and general depression have now been several times recorded after chloralose, but the risk to life seems small owing to the heart and respiration not being profoundly depressed.

Literature, Hanriot, and Richet, Pharmac. Journal, 3 ser. xxiii., 609. Chambard, Revue de Méd., 1894. Cappelletti, Therap. Gazette, Sept., 1894. L'Hoest, Brit. Med. Journal, Oct. 13, 1894. Flemming, Practitioner, July, 1894. Sacaze, Med. Week, Sept. 14, 1894.

